# The Impact of Surgical Treatment with Adjuvant Chemotherapy for Ovarian Cancer on Disorders in the Urinary System and Quality of Life in Women

**DOI:** 10.3390/jcm11051300

**Published:** 2022-02-27

**Authors:** Marcin Opławski, Beniamin Oskar Grabarek, Agata Średnicka, Justyna Czarniecka, Agata Panfil, Zbigniew Kojs, Dariusz Boroń

**Affiliations:** 1 Department of Gynecology and Obstetrics with Gynecologic Oncology, Ludwik Rydygier Memorial Specialized Hospital, 31-826 Kraków, Poland; bgrabarek7@gmail.com (B.O.G.); agata.srednicka@gmail.com (A.Ś.); zkojs@interia.pl (Z.K.); dariusz@boron.pl (D.B.); 2 Department of Gynecology and Obstetrics, Faculty of Medicine and Health Sciences, Andrzej Frycz Modrzewski University in Cracow, 30-705 Cracow, Poland; 3 Department of Histology, Cytophysiology and Embryology in Zabrze, Faculty of Medicine in Zabrze, University of Technology, Academy of Silesia in Katowice, 41-800 Zabrze, Poland; juscza1f@gmail.com (J.C.); agatapanfil@o2.pl (A.P.); 4 Department of Gynecology and Obstetrics in Zabrze, Faculty of Medicine in Zabrze, University of Technology, Academy of Silesia in Katowice, 41-800 Zabrze, Poland

**Keywords:** ovarian cancer, chemotherapy, surgical treatment, urogynecology examination, quality of life, sexuality

## Abstract

Ovarian cancer is the fourth-most-common cause of death among all malignant cancers in women in Poland. This study aimed to compare the functioning of the urinary system and quality of life in women in the 12-month period following the completion of surgery or adjuvant treatment for ovarian cancer, with patients who underwent a hysterectomy for non-oncological reasons (control group). The study group consisted of 50 patients diagnosed with stage I–III ovarian cancer. Among 38 patients with type II ovarian cancer (group A), surgery followed by first-line chemotherapy was performed. Within this group of patients, 20 had stage I ovarian cancer, while 18 had stage II ovarian cancer. The study was performed at least 6 months after the final chemotherapy cycle, with no clinical, marker or radiological recurrence determined. On the other hand, in 12 patients with stage I type I ovarian cancer, oncological treatment consisted of only surgery, without the need for adjuvant chemotherapy, due to the low stage of the lesions (group B). In turn, the control group consisted of 50 women who underwent uterine removal for non-oncological reasons (group C). The assessment of quality of life was conducted using the questionnaires: Satisfaction with Life Scale (SWLS); Incontinence Impact Questionnaire, short form (IIQ-7); Urogenital Distress Inventory (UDI-6); and the Sexual Satisfaction Scale for 3, 6, 9, and 12 months after the conclusion of oncological treatment. During the follow-up, a significant reduction in the quality of everyday life and sexual life was noted among patients with ovarian cancer, more pronounced in group B, compared to the control group (*p* < 0.05). The risk of urinary incontinence is independent of the treatment regimen chosen for ovarian cancer. It is necessary to consider comprehensive psychological care and sexual therapy in patients with ovarian cancer and their families.

## 1. Introduction

According to the National Cancer Registry (NCR), in 2020, ovarian cancer morbidity in Poland was the second-highest as to the frequency of occurrence among cancers of the reproductive organs—3864 cases, after endometrial cancer, with 6659 cases. Moreover, it constitutes the fourth-most-common cause of death among all malignant cancers in women in Poland [1]. In 2018, 295,000 new ovarian cancer cases were diagnosed, leading to the deaths of more than 180,000 women worldwide [2]. Due to its molecular basis, growth type, and prognosis, two types of ovarian cancer can be distinguished [3]. Type I constitutes 30% of all ovarian cancer cases, developing based on benign ovarian tumors or borderline malignancy cancers. It grows in the form of a clear tumor in the ovary, and after some time, it spreads to the peritoneal cavity. Histologically, it is usually endometrial or mucinous, having a slightly better prognosis than type II. Often, it is detected at stages I and II. Type II is more common, developing as malignant from the beginning. Often, a clear tumor is not observed in the ovary, and the disease quickly spreads throughout the peritoneal cavity. Histologically, it is usually a poorly differentiated serum carcinoma with a poor prognosis. Often it is only detected at stages III and IV. Despite its name, ovarian cancer is not just a disease of the ovaries—in most cases, so-called peritoneal dissemination is observed, which is the presence of tumors in other abdominal organs [4].

The 5-year survival rate for stages I and II, known as early-stage ovarian cancer, is about 90%. However, in stage III and IV cases, also known as late-stage ovarian cancer, only 20–40% of patients live beyond five years [5].

Due to a lack of characteristic symptoms, ovarian cancer is usually diagnosed in its late stages. The primary treatment for ovarian cancer is surgical treatment, with complementary chemotherapy. Primary surgical treatment is of the greatest importance to successful treatment, in which complete cytoreduction should be the aim, wherein there are no macroscopically visible changes in the peritoneal cavity; treatment often also concerns resection in the upper level of the abdominal cavity, in more advanced cases often involving multiple intestinal resections or removal of the spleen [6]. The complementary treatment is usually chemotherapy, often supplemented with immunotherapy [7]. Platinum compounds with toxoids are used in first-line chemotherapy. Despite the radicalization of surgical treatment and the introduction of new chemotherapeutic agents, the treatment results are not satisfactory, and any progress is mainly due to extension of the time free from relapse [8]. Side effects are associated with any treatment of cancer patients. In the case of ovarian cancer combined therapy, these include disorders of the gastrointestinal tract, as well as of the nervous, hematopoietic, and urinary systems [9]. The most common urinary system ailments include stress urinary incontinence (SUI), urge urinary incontinence (UUI), and mixed urinary incontinence (MUI). To analyze the quality of life in patients with impaired micturition, the following questionnaires were used: Satisfaction with Life Scale (SWLS); Incontinence Impact Questionnaire, Short Form (IIQ-7); and the Urogenital Distress Inventory (UDI-6). Long and complicated treatment has its consequences on the mental health of patients, self-perception, and the ongoing disease [10]. Additionally, patients also experience other unpleasant ailments from the genitourinary and reproductive systems as a consequence of the cancerous disease itself, as well as the treatment used, including abdominal discomfort, vaginal dryness, hot flushes, and sexual discomfort [11]. Importantly, in most cases, more than one symptom occurs at the same time, often all of them simultaneously and they are related to each other, although they may have different etiologies [12]. Consequently, concomitant symptoms reduce quality of life and functioning in women with cancer [13].

The aim of this study is to compare the functioning of the urinary system in women and their quality of life in the 12-month period after the end of surgery or adjuvant treatment for ovarian cancer, with patients after the removal of the uterus with appendages for non-oncological reasons (control group).

## 2. Materials and Methods

This study was conducted according to the guidelines of the Declaration of Helsinki and approved by the Institution of the Bioethical Committee, operating at the Medical Higher School in Opole, Poland, no. 60/PO/2019 and the Institution of the Bioethical Committee, operating at the Regional Medical Chamber in Krakow, Poland, no. 300/KBL/OIL/2020. Data confidentiality and patient anonymity were maintained at all times. Patient-identifying information was deleted before the database was analyzed. It is not possible to identify patients on an individual level, either from this article or in the database. Informed consent was obtained from all subjects involved in the study. The survey was conducted from 1 June 2017 to 1 September 2021, whereas the operations were conducted in the period of 1 March 2017 to 1 September 2020. This study was conducted on patients treated surgically in the Department of Gynecology and Obstetrics with Oncological Gynecology of the Ludwik Rydygier Hospital in Krakow.

### 2.1. Subjects

The study group in this study consisted of a total 50 patients diagnosed with stage I-III ovarian cancer. Among 38 patients with type II ovarian cancer (group A), surgery followed by first-line chemotherapy, in accordance with the standards, was performed. Within this patient group, 20 had stage I ovarian cancer (age 55.03 ± 4.11 years; BMI 27.06 ± 4.24—overweight), while 18 had stage II ovarian cancer (age 55.66 ± 4.82 years; BMI 28.16 ± 4.55—overweight). The examination was performed at least 6 months after the final chemotherapy cycle, with no clinical, marker or radiological recurrence RECIST determined. In 12 patients with type I, stage I ovarian cancer, (age 55.03 ± 4.11 years; BMI 27.06 ± 4.24—overweight), oncological treatment was performed, which consisted of surgery without the need for adjuvant chemotherapy, due to the low degree of advancement of the changes (group B). In turn, the control group consisted of 50 women (age 55.03 ± 4.11 years; BMI 27.06 ± 4.24—overweight), in whom a hysterectomy was performed for non-oncological reasons (group C). In all study groups, examinations were performed after 3, 6 and 12 months from the end of treatment.

The treatment of all patients was conducted at the Ludwik Rydygier Specialist Hospital in Krakow. Surgery was performed at the Department of Gynecology and Obstetrics with Oncological Gynecology, with adjuvant therapy in the Clinical Oncology Department. As part of the surgical treatment, the uterus with appendages, appendix, net, small pelvic and preaortic lymph nodes were removed. Patients with complete cytoreduction as a result of surgical treatment were included in the study.

### 2.2. Urogynecological Examination

Urogynecological examination was only conducted as a supplementary examination to refine the diagnosis. We decided to use Valsalva Leak Point Pressure values (VLPP values), known as the lowest, critical intra-abdominal pressure, obtained during the Valsalva maneuver, where leakage occurs. The maximum coil closing pressure to confirm SUI was confirmed to be VLPP > 60 cm H_2_O. Urogynecological examination involves: (1) uroflowmetry—urethra flow measurement; (2) post-micturition residual assessment —PVR including cystometry and voiding cystometry; (3) profilometry. The exact urogynecological examination protocol was described by us in a previous study [14].

Every woman from groups A, B, and C was asked about completing the following questionnaires: the SWLS, in which the mental health of the patients was reviewed, IIQ-7, and UDI-6. The results of the urinary tract examinations were interpreted as 4 possible diagnoses:

1. No symptoms connected with the urinary system;
2. Stress urinary incontinence (SUI);
3. Overactive bladder (OB);
4. Mixed Urinary Incontinence (MUI).

### 2.3. Quality of Life’s Aspects Assessment

#### 2.3.1. The Satisfaction with Life Scale

The Satisfaction with Life Scale (SWLS) is utilized for self-assessment of life satisfaction, related to mental health, subjective quality of life and the likelihood of suicide attempts. Respondents give answers to 5 questions, measured according to a 7-point scale, where 1 signifies “definitely disagree” with the statement, and 7 signifies “definitely agree” with the statement. This test has high internal consistency and test-retest correlations [15].

After assigning a value from 1 to 7 to each of the statements and adding up the points, it is possible to determined overall life satisfaction. The higher the score, the higher the sense of satisfaction with life.

The obtained score should be interpreted in the following way [16]:

- 31–35 = Very satisfied

Such a high score indicates that the respondent is really satisfied with their life and feels that everything is going very well. This does not indicate that their life is perfect, but that they are satisfied with what it looks like, and that challenges are temporary and solvable.

- 26–30 = Satisfied

The respondent feels that most things in their life are going very well but there may be one or two key areas which they want to change.

- 21–25 = Somewhat satisfied

This score indicates that the respondent is generally satisfied on a daily basis; however, there are areas they would really like to improve.

- 20 = Neutral

This score indicates that the respondent does not think too much about what does and does not require improvement and is quite satisfied with the current situation.

- 15–19 = Somewhat unsatisfied

This score indicates that the respondent feels more unsatisfied than satisfied on a daily basis, and several key areas require improvement. This may also indicate that there is general satisfaction but there is one area of life in which they are deeply unsatisfied.

- 10–14 = Unsatisfied

This score indicates that the respondent feels seriously unsatisfied with the current situation. This may be deep dissatisfaction in all areas of life or there are two or three areas which are far worse than others.

- 5–9 = Very unsatisfied

This score indicates extreme dissatisfaction with the current life situation in many areas of life.

In our paper the SWLS survey on quality of life was assessed for 3 possible situations:

1. State unchanged (20 points)
2. Decrease in quality of life (1–19 points)
3. Increase in quality of life (21–35 points)

#### 2.3.2. Assessment of Chronic Illness Therapy-Fatigue

The 13-part questionnaire, Functional Assessment of Chronic Illness Therapy-Fatigue (FACIT-F) was utilized to assess fatigue levels during everyday tasks in the week prior to the questionnaire being completed by patients of the three groups (A, B, C). Each statement is assessed on a 4-point Likert scale (4 = not at all fatigued, to 0 = very much fatigued) with acceptable reliability, validity, and sensitivity to changes over time [17]. Thanks to the scale, the influence of the disease on 4 aspects of life can be assessed: (1) physical well-being—7 questions, maximum 28 points; (2) social/family well-being—7 questions, maximum 28 points; (3) emotional well-being—6 questions, maximum 24 points; (4) functional well-being—7 questions, maximum 28 points; (5) fatigue subscale—13 questions, maximum 52 points.

The following average values were adopted for the tested scales: (1) physical well-being—a value above 14 points being a good satisfactory result; (2) social/family well-being—>15 points; (3) emotional well-being—>13 points; (4) functional well-being—>14 points; (5) fatigue subscale—>26 points. FACIT-F determines the overall quality of life of the respondents in particular aspects of life, with an acceptable value assumed at >80 points. The higher the numerical value, the higher the quality of life.

#### 2.3.3. Assessment of the Pain Level

**Figure 1:**
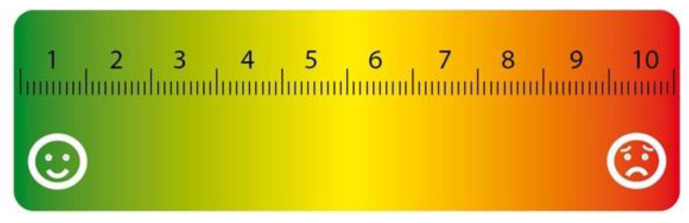
Visual Analog Scale—a special (colored) ruler for pain assessment [own drawing].

Furthermore, pain intensity was assessed in all patients using the Visual Analog Scale (VAS) in the form of a line on which the examined individual indicated the intensity of pain on a scale of 0 to 10, where 0 signified no pain and 10 signified intense pain (Figure 1). The numerical VAS value indicated by the patient was recorded in the database.

#### 2.3.4. The Sexual Satisfaction Scale (SSS)

The level of self-assessed sexual satisfaction was measured using Davies’ Sexual Satisfaction Scale (SSS), translated into Polish by Szumski and Małecki. The scale consists of 21 statements, answered by the examined individuals using a five-point Likert scale (1—Strongly Disagree; 2—Disagree; 3—Undecided; 4—Agree; 5—Strongly Agree), with the obtained score in the range of 21 to 105 [18].

The questionnaire comprised of three subsections:

- Physical sexual satisfaction—relating to the assessment of the quality of sexual contact in a relationship, the sexual abilities of the partner, and the satisfaction of the individual’s needs in the relationship. The obtained score was in the range of 11 to 55.
- Emotional sexual satisfaction—measuring the affective feelings towards sex and the partner’s behavior, as well as their feelings towards their partner. The obtained score was in the range of 4 to 20.
- Control-related sexual satisfaction—relating to the assessment of their own influence over, when, and if at all the individual has sexual contact. The obtained score was in the range of 6 to 30.

### 2.4. Statistical Analysis

Statistical analysis was performed using the licensed version of the STATISTICA 13.0 (StatSoft, Cracow, Poland) software. The Chi-square test (Chi2) was used to analyze the relationship between the qualitative characteristics. The level of *p* < 0.05 was adopted as the statistical significance threshold. The results of the test were compared between groups: group A vs. C; group B vs. C; group A vs. B. In the final stage, the Pearson correlation analysis between the questionnaires—IIQ-7 and UDI-6—determined the type of urinary incontinence, alongside results from the urogynecological examination (*p* < 0.05).

## 3. Results

### 3.1. The Quality of Life in Patients with Impaired Micturition the Questionnaires UDI-6, II-Q7

Firstly, the frequency of occurrence of the following forms of urinary incontinence was analyzed: stress urinary incontinence, overactive bladder, and mixed urinary incontinence among patients after 3, 6, 9 and 12 months from the conclusion of ovarian cancer treatment (groups A and B) compared to a group of patients who had had a hysterectomy for non-oncological reasons (group C). The smallest influence on the functionality of the urinary system can be observed in the control group, where the number of patients not reporting any urinary system dysfunction ranges from 40–43 patients (80–86%). In this group, the most-commonly diagnosed urinary system dysfunction was stress urinary incontinence (3–6 patients; 6–12%). In turn, in group A, the most commonly occurring urinary system dysfunction was noted to be an overactive bladder (18–21 patients; 47.36–55.26%). It should also be noted that, as time elapsed from the end of surgical treatment and chemotherapy, the number of patients without a dysfunction also increased (12–16 patients; 31.58–42.11%). In turn, in group B, where ovarian cancer treatment consisted of only surgery, the number of patients in whom, during the urogynecological examination, no dysfunction was reported was 7–9 patients (58.33–75%). Regardless of the study period, statistically significant differences were observed when comparing characteristics between the groups: A vs. C; B vs. C; A + B vs. C; as well as between groups A and B (Table 1, *p* < 0.05).

### 3.2. The Satisfaction with Satisfaction Life Scale

Next, based on the SWLS questionnaire, the quality of life of the three groups analyzed as part of this study was assessed. In the control group, regardless of the time elapsed from the end of surgical treatment. No quality-of-life changes were declared by 27–30 patients (54–60%). Alongside this decrease in the number of women in whom treatment did not affect quality of life or cause deterioration in group C, there was an increase in the number of patients who reported an improvement (15–22 patients; 30–44%).

In contrast, in the study groups A and B, a deterioration in quality of life could be observed in the assessed post-operative period, with a larger number of these patients being in group A. In the group of patients where surgical treatment was supplemented with chemotherapy (group A), 15–18 patients (39.47–55.26%) declared no effect of the therapy on the quality of treatment, while 18-21 patients noted a deterioration in their quality of life after treatment (47.37–55.26%). In turn, among patients where ovarian cancer treatment was limited to only surgical treatment, a lack of change in quality of life was reported by 5 (41.67%), improvement was reported by 5–7 women (41.67–58.33%), and a deterioration by 0–2 patients (0–16.67%).

Statistical analysis indicated the presence of statistical significance at each time point between the groups: A vs. C; B vs. C; as well as A + B vs. C (*p* < 0.05; Table 4).

### 3.3. Functional Assessment of Chronic Illness Therapy-Fatigue (FACIT-F)

Next, we assessed the effects of treatment in individual groups on the level of perceived fatigue in the period from 3 to 12 months after the end of treatment. Table 5 shows the total score obtained from the responses to the FACTI-F questionnaire. The interpretation of the results showed that, in the control group only, up to 3 months after the surgery, the patients experienced fatigue, but their overall quality of life did not deteriorate (mean sum of 91.27 points). In the remaining periods, no fatigue was noted among women in the control group (score over 30). In group A, regardless of the time that elapsed from the end of the therapy, it can be stated that the patients experienced fatigue and its level decreased over time. In this group, the lowest total FATIC-F score was also noted, regardless of the period from the end of treatment. Even after 12 months, the overall quality of life was below satisfactory (<80 points). Furthermore, in group B within 9 months from the surgery, the obtained result indicates that the patients felt tired. In the 12th month of observation, the result concerning the area of fatigue tends to be satisfactory (>30 points), although not in all patients of group B. Statistical analysis showed statistical significance at each time point between groups A vs. C, B vs. C and A + B vs. C (Table 5; *p* < 0.05).

### 3.4. Assessment of the Pain Level

Subsequently, the perception of pain by the patients of groups A, B and C within 3–12 months of the end of treatment was assessed. The most severe pain symptoms were reported by the patients of group A, especially within 3 months of the end of treatment. After 12 months, a result similar to that declared by the control group can be recorded. In addition, in group B the level of perceived pain was higher than in the control group, but lower than in group A. Statistical analysis showed statistical significance between groups A vs. C, B vs. C and A + B vs. C (Table 6; *p* < 0.05).

In the last stage, the influence of the applied therapy on sexual satisfaction was assessed. The higher the score, the higher the patient’s satisfaction with their sex life. The lowest satisfaction with their sex life was reported by patients of group A, and in months 9 and 12, the level of dissatisfaction increased significantly compared to the previous period. In addition, comparing the obtained results with the control group, in group B the treatment also had a negative effect on sexual life, but to a lesser extent than in group A. Statistical analysis showed the presence of statistical significance at each time point between groups A vs. C, B vs. C and A + B vs. C (Table 7; *p* < 0.05).

## 4. Discussion

Ovarian cancer is not one of the most common gynecological cancers, but it is characterized by a high mortality rate [19], therefore limiting the normal functioning of women in everyday life, forcing them to change their lifestyle [20].

One of the more commonly occurring side-effects of combination therapy for ovarian or endometrial cancer is the occurrence of urinary system dysfunction in the form of incontinence disorders [21,22,23,24,25].

We conducted our study at least 6 months after the last cycle of chemotherapy in women in whom there was no clinical, marker or radiological recurrence of RECIST.

Urinary system dysfunction negatively influences the quality of life of the patients of both study groups compared to the control group. Our results confirm the observations of Neron et al. [26], who found that, in the group of women with a neoplastic history of endometrial or ovarian cancer, the incidence of negative pelvic floor and urinary incontinence symptoms as well as reduced quality of life is significantly higher than in the control group. An important supplement to these observations was the analysis conducted by Ramaseshan et al. [27] in 2017, who also showed that disorders in the pelvic and genitourinary tract in women with oncological cancer, and thus a lower quality of life, are more common in cancer patients. Cascales-Campos et al. [28] found that, among patients with advanced ovarian cancer 12 months after the end of treatment, 45% had urinary incontinence, 20% had fecal incontinence, and 14% had simultaneous urinary and fecal incontinence. In our study, the percentage of women struggling with urinary incontinence in group A was analogous to that described by Cascales-Campos et al. [28], ranging from 58% to 65%, a higher prevalence, possibly resulting from, inter alia, a different group size, as well as Cascales-Campos et al. not conducting urogynecological examinations of patients. Another potential reason explaining the higher percentage of women with urinary incontinence after ovarian cancer treatment in the Polish population compared to the Spanish population, as described by Cascales-Campos et al. may be due to the fact that, in Spain, a low percentage of women with no oncological history struggling with urinary incontinence was found—23%, while in France it is 43% [29], and in Poland, based on a study by Przydacz et al. [30], this problem affects 21.3–36.6% of women. The reliability of the results obtained from the conducted questionnaire studies can be compared with the studies conducted in 2013–2014 at the Świętokrzyskie Oncology Center in Kielce. The study used the European Organization for Research and Treatment of Cancer QOL Core Questionnaire 30 (EORTC QLQ-30), Beck’s Depression Self-Assessment Scale and a socio-demographic-medical questionnaire. It was shown that the quality of life of depressive patients was statistically significantly different from that of patients without features of depression. A statistical correlation was confirmed between the severity of depression and the assessment of the quality of life of patients diagnosed with cancer of the reproductive organ. With the passage of postoperative time, the quality of life and the degree of life satisfaction gradually increased, thus reducing the level of depression in the respondents [31].

Next, we assessed the effect of the treatment on the degree of fatigue experienced by the patients using the FACIT-F questionnaire and the intensity of pain via the VAS scale. It should be remembered that the FACIT-Fi questionnaires and the VAS scale are used successfully outside of oncology, too [32,33].

The obtained results confirm the participation of cancer itself in the feeling of fatigue (in group C, it was felt for only 3 months, and in group B for 6 months), as well as the significant influence of adjuvant chemotherapy on the experienced fatigue (fatigue was felt throughout the 12-month observation period). Levesque et al. [34] conducted questionnaire studies in a group of 36 women with ovarian cancer. Based on the obtained results, they concluded that the neoplastic process significantly influences the psychological wellbeing of the patients. These studies confirm that oncological therapy cannot end with the treatment related to the removal of the lesion alone, but must also consider the psychological health of the patient and her family [35]. In turn, Deborah et al. [35] used the FACIT-H questionnaire in a group of six women with breast cancer and three with ovarian cancer, who participated three times a week for eight weeks in the EU fitness boxing program. Following this, 22% of respondents reported a reduction in pain from “little” to “not at all”, and about 44% reported a reduction in their concern about the side effects of treatment from “little” to “not at all”; increase in good sleep from “little” to “very much”; and reduction in fatigue from “a little” to “not at all” [35]. It should be noted that this study was performed on a small population, hence its value is negligible. Okada et al. [36] assessed the level of perceived fatigue in a group of pancreatic cancer patients treated with nab-paclitaxel plus gemcitabine. Their observations show the legitimacy of using the FACIT-F questionnaire among cancer patients. The results obtained by these researchers are much lower than ours (0.2–1.6), which may be due to a different type of cancer, as well as the assessment of the FACIT-F questionnaire during chemotherapy. In addition, the analysis by Meregialgia et al. [37] indicates that the FACIT-F questionnaire may come from EuroQol 5-Dimensions 5-Levels (EQ-5D-5L) utilities from Functional Assessment of Anorexia-Cachexia Therapy (FAACT) and Functional Assessment of Cancer Therapy-General—FACT-G, a useful tool in the economic analyses of the treatment of patients with lung cancer as well as other cancers.

Therefore, considering the results presented so far, it is possible to notice not only a significant reduction in the quality of life, but also a lack of willingness to live and perform daily duties, disturbance in contacts with the environment, or anhedonia. This emphasizes the importance of providing patients with psychological care throughout the treatment period and after its completion. In the collected interviews, there is no information about using psychological or psychiatric help or taking antidepressants, which is one of the points that should be considered in our further research.

In the last stage of the research, we assessed the influence of the treatment used for self-esteem and satisfaction with sexual life.

Research by Fisher et al. [38] among women with ovarian cancer, based on six questionnaires (SSS was not used), confirmed a worse quality of life for these women, greater sexual dysfunction and sexual distress compared to the population norms of healthy women. These women are characterized by a change in their relationship satisfaction, feelings of anxiety, resentment, anxiety, and fear. Patients in this study describe pain during intercourse, no orgasm, or a shift to a shorter one that requires a longer time to achieve. The physicality of a woman is also important. Sexual disorders are also influenced by the cultural context in which the uterus and ovaries are symbols of women and femininity [38].

It can be said with high probability that the results concerning quality of life presented by us include not only disease and urinary incontinence, but also sexual dysfunction and the disturbance of the image of one’s body and sexuality by the disease.

Ovarian cancer can cause a fear of being approached, touched, committed to a relationship, and meeting the other party’s expectations. Juraskova et al. [39] stated that patients with cervical and endometrial cancer, after the end of treatment, prioritized their partner’s sexual needs over their own and that they had a strong need to provide their partner with intercourse despite their own difficulties and sexual disorders. They view sex as a duty in a relationship/marriage.

When analyzing the results concerning sexuality in cancer patients/women, the type of cancer should be considered. People with hematological tumors [40] or breast cancer [41] had fewer sexual disorders than those presented by us or Juraskova et al. [39]. It should also be remembered that hysterotomy performed for non-oncological reasons or unrelated to benign neoplasms does not have the same negative effects on sexuality as a neoplasm. However, it has not yet been established whether ovariectomy for benign and malignant lesions has different sexual implications.

Stafford et al. [41], using a multivariate model and demographic, medical and psychosocial predictors, showed that married women, under 56 years of age and untreated, accepted their appearance and, with a longer period of time from diagnosis, were significantly more sexually active [41]. That is why it is so important to raise the qualifications of medical personnel in discussing physical and sexual changes related to the proposed and necessary oncological treatment [42]. Interestingly, Mayer et al. [43] in a study of 396 women with breast cancer and 93 women with ovarian cancer, noted that within a period of at least 24 months from receiving the diagnosis, the patients did not declare any effect on sexual activity and quality of life regardless of the type and radicality of the procedure or chemotherapy in patients with breast and ovarian cancer, which is contrary to our observations [43]. However, the cited observation of Mayer et al. [43] seems to be isolated in the light of our and other researchers’ observations. Akhert et al. [44] clearly indicate that the quality of life of patients with ovarian cancer is low [44].

Following the results obtained by us regarding the quality of life of patients with ovarian cancer who are receiving appropriate treatment depending on the stage of the lesions, it seems important not only to provide appropriate psychological care, but also to search for new forms of chemotherapy and strategies to improve the effectiveness of therapy [45,46]. We can expect their results in the next few years.

Of course, our study has several limitations. The first of these may be the relatively small size of the individual groups. However, it should be remembered that this is a single-center study, while ovarian cancer is not the most common gynecological neoplasm in women and is characterized by low detection rates [1]. In addition, only some of the available tools to assess the quality of life of cancer patients were used. For this reason, further analyses seem necessary, and the results obtained so far seem valuable.

## 5. Conclusions

In summary, one universal questionnaire, on the basis of which any form of urinary incontinence can be determined, does not exist. Studies regarding quality of life and sexuality indicate that both decrease with ovarian cancer, even more significantly in the case of surgical treatment with adjuvant chemotherapy. Therefore, considering the current standards of gynecological and oncological care in Poland, as well as in other countries undoubtedly, the psychological care of patients, in addition to that of the family in which gynecological cancer has occurred, should be unrestricted and be a routine medical procedure.

## Figures and Tables

**Table 1:** Comparison of urogynecological examination in the study groups.

Time from the Completion of Treatment (Months)	Result of Treatment	N			*p*-Value
		Group C	Group A	Group B	
3	No changes (NC)	40	14	7	0.0174 *
	Stress urinary incontinence (SUI)	6	1	1	0.0078 **
	Overactive Bladder (OAB)	2	20	2	0.0212 ***
	Mixed urinary incontinence (MUI)	2	3	1	0.0899 ****
				0	0.0492
6	No changes (NC)	41	12	8	0.0155 *
	Stress urinary incontinence (SUI)	5	1	1	0.0076 **
	Overactive Bladder (OAB)	2	21	1	0.0019 ***
	Mixed urinary incontinence (MUI)	2	4	1	0.3450 ****
9	No changes (NC)	41	15	8	0.0111 *
	Stress urinary incontinence (SUI)	5	1	1	0.0052 **
	Overactive Bladder (OAB)	3	18	1	0.0287 ***
	Mixed urinary incontinence (MUI)	1	4	1	0.0099 ****
12	No changes (NC)	43	16	9	0.0177 *
	Stress urinary incontinence (SUI)	3	1	1	0.0023 **
	Overactive Bladder (OAB)	3	17	1	0.0123 ***
	Mixed urinary incontinence (MUI)	1	4	0	0.0190 ****

* C vs. A, ** C vs. B, *** A vs. B, **** C vs. (A + B)—statistically significant differences (*p* < 0.05). In the next stage, the usefulness of utilizing the UDI-6 and IIQ-7 questionnaires in certain types of urinary incontinence was assessed. For this purpose, a correlation analysis was performed between the type of urinary incontinence determined through the questionnaires and the results of the urodynamic examination. The correlation coefficients for the types of urinary incontinence were observed as follows: stress urinary incontinence—r = +0.71 (*p* < 0.05); neurogenic bladder—r = +0.26 (*p* > 0.05); mixed urinary incontinence—r = +0.77 (*p* < 0.05). For the UDI-6 questionnaire, the following interdependencies were determined: stress urinary incontinence—r = +0.92 (*p* < 0.05); neurogenic bladder—r = +0.45 (*p* > 0.05); mixed urinary incontinence, it was noted to be r = +0.32 (*p* < 0.05). The obtained results for the UDI-6 and IIQ-7 questionnaires were presented in Table 2 and Table 3.

**Table 2:** Results of the UDI-6 survey.

Time from the Completion of Treatment (Months)	Answer Variant	Do You Urinate Frequently?			Do You Find There Is Leakage of Urine Associated with the Feeling of Pressure on the Bladder?			Do You Find There Is Leakage of Urine Due to Physical Activity, Coughing, or Sneezing?			Do You Find There Is Leakage of Urine in Small Amounts (Droplets)?			Do You Have Problems with Emptying Your Bladder?			Do You Have Pain in the Lower Abdomen or around the Vulva?		
		C	A	B	C	A	B	C	A	B	C	A	B	C	A	B	C	A	B
3	No	38	15	7	35	33	5	35	33	4	40	33	7	42	35	5	35	33	7
	Not at all	4	0	1	5	0	2	5	0	2	5	0	2	4	0	5	5	0	3
	Some/A little	4	3	1	5	3	2	5	1	3	2	1	1	2	2	1	5	3	1
	Medium	2	12	2	3	1	2	3	2	2	2	1	1	1	1	1	3	2	1
	Very/A lot	2	8	1	2	1	1	2	2	1	1	3	1	1	0	0	2	0	0
6	No	39	13	7	36	34	5	37	33	4	40	33	7	43	36	5	35	35	7
	Not at all	4	0	2	5	0	3	5	0	3	5	0	3	4	0	5	5	0	3
	Some/A little	3	7	1	4	2	1	3	1	2	3	0	1	2	1	1	7	2	1
	Medium	2	10	1	3	1	1	3	1	1	2	2	1	1	1	1	2	1	1
	Very/A lot	2	8	1	2	1	1	2	3	1	0	3	0	0	0	0	1	0	0
9	No	40	16	8	38	33	33	39	32	5	41	33	7	44	36	6	35	35	7
	Not at all	4	0	1	5	0	0	5	0	3	5	0	4	4	0	5	5	0	4
	Some/A little	3	8	1	4	3	3	3	2	1	2	1	1	1	2	1	8	3	1
	Medium	2	10	2	2	1	1	2	1	1	2	1	0	1	0	0	2	0	0
	Very/A lot	1	4	0	1	1	1	1	3	1	0	3	0	0	0	0	0	0	0
12	No	41	17	8	39	34	34	40	34	6	43	33	7	4	36	6	37	36	7
	Not at all	3	0	2	5	0	0	5	0	4	5	0	4	4	0	5	5	0	4
	Some/A little	3	8	1	3	1	1	3	1	1	1	1	1	4	2	1	6	2	1
	Medium	2	9	1	2	1	1	2	2	1	1	2	0	0	0	0	2	0	0
	Very/A lot	1	4	0	1	1	1	0	1	0	0	2	0	0	0	0	0	0	0

**Table 3:** Results of IIQ-7 survey.

Time from the Completion of Treatment (Months)	Answer Variant	Does Leakage of Urine and/or Lowering/Prolapse of the Vaginal Walls/Reproductive Organ Affect You in the Following Categories:																				
		Ability to Do Household Activities			Physical Recreation Such as Walking, Swimming			Entertainment, Such as Going to the Cinema or a Concert			Possibility to Travel by Car or Bus for Longer than 30 Min from the House			Participation in Social Activities outside the Home			Mental/Emotional Health			Feeling Frustrated		
		C	A	B	C	A	B	C	A	B	C	A	B	C	A	B	C	A	B	C	A	B
3	No	40	18	10	40	20	10	45	23	11	38	28	10	39	25	11	42	15	7	46	11	6
	Not at all	0	0	0	2	0	0	2	0	0	5	0	0	2	0	0	6	0	2	3	0	3
	Some/A little	5	12	2	5	12	2	2	13	1	5	7	2	6	12	1	1	5	3	1	9	1
	Medium	3	5	0	2	5	0	1	2	0	2	3	0	2	1	0	1	10	0	0	10	2
	Very/A lot	2	3	0	1	1	0	0	0	0	1	0	0	1	0	0	0	8	0	0	8	0
6	No	40	21	10	40	21	10	45	25	11	38	29	11	39	27	11	42	16	7	46	13	5
	Not at all	0	0	1	2	0	1	4	0	0	6	0	0	4	0	0	6	0	3	3	0	3
	Some/A little	6	12	1	6	12	1	1	12	1	5	7	1	5	10	1	1	8	2	1	8	2
	Medium	3	5	0	2	5	0	0	1	0	1	2	0	2	1	0	1	5	0	0	8	2
	Very/A lot	1	0	0	0	0	0	0	0	0	0	0	0	0	0	0	0	9	0	0	9	1
9	No	40	25	10	40	23	11	45	25	11	38	29	11	49	27	11	44	18	7	48	14	6
	Not at all	0	0	1	5	0	1	4	0	0	8	0	0	5	0	0	5	0	3	1	0	3
	Some/A little	7	11	1	3	12	0	1	13	1	3	8	1	4	11	1	1	10	2	1	9	3
	Medium	3	2	0	2	3	0	0	0	0	1	1		2	0	0	0	5	0	0	9	0
	Very/A lot	0	0	0	0	0	0	0	0	0	0	0	0	0	0	0	0	5	0	0	6	0
12	No	40	26	10	40	22	11	45	27	11	38	29	11	39	29	11	44	16	9	1	19	6
	Not at all	4	0	1	17	0	1	4	0	0	9	0	0	7	0	0	5	0	2	1	0	3
	Some/A little	5	11	1	3	11	0	1	11	1	3	9	1	4	9	1	1	10	0	0	10	3
	Medium	1	1	0	0	5	0	0	0	0	0	0	0	0	0	0	0	6	0	0	6	0
	Very/A lot	0	0	0	0	1	0	0	0	0	0	0	0	0	0	0	0	6	0	1	3	0

**Table 4:** Results of the SWLS survey assessing the quality of life.

Time from the Completion of Treatment (Months)	Result of Treatment	N			*p*-Value
		Group C	Group A	Group B	
3	No changes	30	15	5	0.0014 * 0.0055 ** 0.8642 *** 0.0002 ****
	Better	15	3	5	
	Worse	5	20	2	
6	No changes	30	16	5	0.0013 * 0.0055 ** 0.8282 *** 0.0002 ****
	Better	18	1	6	
	Worse	2	21	1	
9	No changes	28	18	5	0.0014 * 0.0057 ** 0.8501 *** 0.0002 ****
	Better	20	2	6	
	Worse	2	18	1	
12	No changes	27	16	5	0.0015 * 0.0054 ** 0.8642 *** 0.0019 ****
	Better	22	3	7	
	Worse	1	19	0	

* C vs. A, ** C vs. B, *** A vs. B, **** C vs. (A + B)—statistically significant differences (*p* < 0.05).

**Table 5:** Results of the FACIT-F survey from the study groups and control group.

Time from the Completion of Treatment (Months)	Aspect of Life	Group C	Group A	Group B	*p*-Value
3	total score (sum points)	91.27 ± 2.98	45.09 ± 1.76	67.86 ± 1.22	0.0002 * 0.0008 ** 0.0026 *** 0.0001 ****
	physical well-being	17.98 ± 1.41	9.76 ± 1.25	12.04 ± 2.11	0.0000 * 0.0000 ** 0.0054 *** 0.0000 ****
	social/family well-being	21.43 ± 1.87	7.65 ± 2.09	9.87 ± 1.77	0.0000 * 0.0000 ** 0.0439 *** 0.0000 ****
	emotional well-being	17.88 ± 1.36	10.09 ± 1.56	12.98 ± 1.23	0.0000 * 0.0000 ** 0.0467 *** 0.0000 ****
	functional well-being	11.99 ± 1.09	4.52 ± 1.44	10.99 ± 2.87	0.0000 * 0.0000 ** 0.0000 *** 0.0000 ****
	fatigue subscale	21.99±2.44	13.07±1.56	21.98±1.13	0.0000 * 0.0000 ** 0.0000 *** 0.0000 ****
6	total score (sum points)	112.83 ± 3.67	57.84 ± 3.09	77.12 ± 1.45	0.0000 * 0.0000 ** 0.0000 *** 0.0000 ****
	physical well-being	20.22 ± 2.09	9.54 ± 2.98	13.91 ± 1.56	0.0000 * 0.0000 ** 0.0087 *** 0.0000 ****
	social/family well-being	19.98 ± 1.98	9.89 ± 1.75	12.01 ± 1.48	0.0000 * 0.0000 ** 0.0045 *** 0.0000 ****
	emotional well-being	21.87 ± 2.56	12.99 ± 1.86	11.24 ± 1.34	0.0000 * 0.0000 ** 0.0789 *** 0.0000 ****
	functional well-being	19.99 ± 1.54	10.34 ± 2.12	13.98 ± 1.23	0.0000 * 0.0000 ** 0.0234 *** 0.0000 ****
	fatigue subscale	30.77 ± 2.09	15.08 ± 3.11	25.98 ± 1.11	0.0000 * 0.0000 ** 0.0000 *** 0.0000 ****
9	total score (sum points)	129.28 ± 4.76	67.98 ± 4.13	83.05±1.67	0.0000 * 0.0000 ** 0.0000 *** 0.0000 ****
	physical well-being	21.09 ± 1.93	11.98 ± 1.09	13.76 ± 1.92	0.0000 * 0.0000 ** 0.0412 *** 0.0000 ****
	social/family well-being	22.19 ± 1.76	10.03 ± 1.14	13.45 ± 1.40	0.0000 * 0.0000 ** 0.0399 *** 0.0000 ****
	emotional well-being	22.36 ± 1.87	13.98 ± 2.76	11.09 ± 1.23	0.0000 * 0.0000 ** 0.0456 *** 0.0000 ****
	functional well-being	22.77 ± 1.97	12.01 ± 2.43	16.77 ± 1.45	0.0000 * 0.0000 ** 0.0000 *** 0.0000 ****
	fatigue subscale	40.87 ± 1.56	19.99 ± 2.87	27.98 ± 1.87	0.0000 * 0.0000 ** 0.0000 *** 0.0000 ****
12	total score (sum points)	129.82 ± 4.67	75.5 ± 2.91	101.57 ± 4.99	0.0000 * 0.0000 ** 0.0000 *** 0.0000 ****
	physical well-being	22.13 ± 1.98	12.01 ± 1.99	15.21 ± 1.01	0.0000 * 0.0000 ** 0.0000 *** 0.0000 ****
	social/family well-being	22.18±2.98	14.98±2.09	16.98±1.12	0.0000 * 0.0000 ** 0.0785 *** 0.0000 ****
	emotional well-being	22.98 ± 2.65	14.98 ± 1.65	18.11 ± 1.87	0.0000 * 0.0000 ** 0.0043 *** 0.0000 ****
	functional well-being	22.54 ± 1.43	11.97 ± 1.23	21.19 ± 1.77	0.0000 * 0.0000 ** 0.0000 *** 0.0000 ****
	fatigue subscale	39.99 ± 2.08	21.56 ± 1.66	30.08 ± 1.99	0.0000 * 0.0000 ** 0.0000 *** 0.0000 ****

* C vs. A, ** C vs. B, *** A vs. B, **** C vs. (A + B)—statistically significant differences (*p* < 0.05); mean ± standard deviation.

**Table 6:** Changes in pain levels in the study groups and control group during the 12-month observation.

Time from the Completion of Treatment (Months)	Group C	Group A	Group B	*p*-Value
3	2.46 ± 0.45	5.54 ± 0.75	3.62 ± 0.72	0.0054 * 0.0891 ** 0.0432 *** 0.0089 ****
6	2.11 ± 0.36	4.09 ± 0.56	3.11 ± 0.43	0.0214 * 0.0793 ** 0.1564 *** 0.2315 ****
9	2.43 ± 0.54	2.32 ± 0.65	2.65 ± 2.09	0.7654 * 0.8901 ** 0.9132 *** 0.8967 ****
12	1.76 ± 0.21	2.01 ± 0.87	1.77 ± 0.35	0.9432 * 0.9998 ** 0.9874 *** 0.8782 ***

* C vs. A, ** C vs. B, *** A vs. B, **** C vs. (A + B)—statistically significant differences (*p* < 0.05). mean ± standard deviation.

**Table 7:** Sexual Satisfaction—SSS.

Time after the Completion of Treatment (Months)	C	A	B	*p*-Value
3	67.54 ± 2.90	32.07 ± 2.76	42.09 ± 3.43	0.0013 * 0.0043 ** 0.0165 *** 0.0032 ***
6	69.17 ± 3.45	31.09 ± 3.65	46.09 ± 2.98	0.0012 * 0.0058 ** 0.0241 ** 0.0047 ***
9	71.09 ± 2.12	54.01 ± 4.10	59.11 ± 3.90	0.0076 * 0.0087 ** 0.0165 *** 0.0040 ***
12	79.14 ± 3.07	52.09 ± 3.69	58.92 ± 2.91	0.0087 * 0.0064 ** 0.0305 *** 0.00331 ***

* C vs. A, ** C vs. B, *** A vs. B, **** vs. (A + B)—statistically significant differences (*p* < 0.05); mean ± standard deviation.

## Data Availability

The data used to support the findings of this study are included in the article. The data will not be shared to protect third-party rights and commercial confidentiality.
